# Effect of Family MUAC Utilization in Identifying Severity of Acute Malnutrition at Admission to Nutrition Programs Among Children Aged 6–59 Months Ethiopia

**DOI:** 10.1111/mcn.70054

**Published:** 2025-05-29

**Authors:** Meron Tamirat, Alinoor Mohammed Farah, Gudina Egeta, Aweke kebede, Samson Gebremedhin, Seifu Hagos Gebreyesus

**Affiliations:** ^1^ Departement of Nutrition and Dietetics, School of Public Health Addis Ababa University, College of Health Science Addis Ababa Ethiopia; ^2^ Department of Public Health Nutrition, School of Public Health, College of Medicine and Health Sciences Jigjiga University Jigjiga Ethiopia; ^3^ World Food Program Addis Ababa Ethiopia

**Keywords:** acute malnutrition, caregivers, Ethiopia, family mid‐upper arm circumference

## Abstract

Mid‐upper arm circumference (MUAC) screening is a simple community‐level method for detecting acute malnutrition. The Family MUAC approach, which trains caregivers to measure their children's MUAC and refer them for treatment, has shown promise, but evidence regarding its impact on malnutrition severity at admission is limited. To address this gap, we conducted a longitudinal study from March to May 2024 in two districts in Eastern Ethiopia, enrolling 360 children aged 6–59 months. We compared children referred by their mothers or caregivers using the Family MUAC (*n* = 180) with those referred by Health Extension Workers (HEWs) (*n* = 180). We found that the median MUAC at admission was 119 mm (IQR 116–120) in the mother‐referral group versus 116 mm (IQR 115–119) in the HEWs‐referral group, and the proportion of severe acute malnutrition (MUAC < 115 mm) was lower among caregiver‐referred children (4.2% vs. 18.4%). Multivariable regression analysis showed that mother/caregiver‐referred children had an 80.5% lower risk of severe MUAC at admission [ARR 0.195(0.06, 0.59)] and a 75% reduced likelihood of SAM admission compared to the HEWs‐Referral group (ARR 0.25; 95% CI, 0.148–0.448). The Family MUAC approach significantly reduced the severity of malnutrition at admission. Consequently, this strategy should be expanded and prioritized in national screening programs.

## Summary

- The median mid‐upper arm circumference (MUAC) at admission was higher in Mother‐Referral (119 mm) versus HEWs‐Referral (116 mm).
- Severe acute malnutrition (SAM) was lower among caregiver‐referred children (4.2% vs. 18.4%).
- Multivariable regression showed mother‐referred children had an 80.5% lower risk of severe MUAC at admission and 75% reduced likelihood of SAM admission compared to HEWs‐Referral.
- Wealth status, household size, proximity to health facilities, caregiver's health‐seeking behavior, and absence of recent illnesses influenced malnutrition severity at admission.
- Family MUAC significantly reduced malnutrition severity at admission and should be expanded in national screening programs.

## Graphical abstract

Family mid‐upper arm circumference (MUAC) has demonstrated the ability to enhance early detection of acute malnutrition by identifying children with milder forms of wasting at an earlier stage. Consequently, the use of Family MUAC can significantly improve early case identification and has the potential to improve the coverage of treatment services for wasting or acute malnutrition.

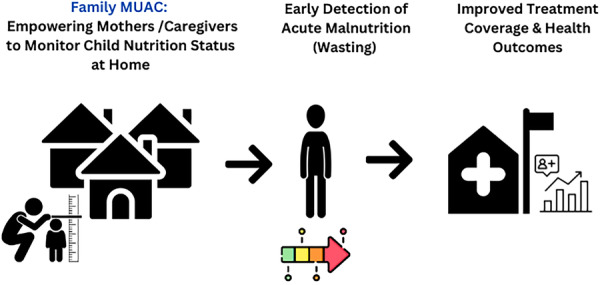

## 1 Introduction

Malnutrition is a major public health concern in many developing countries. Malnutrition weakens the immune system. In addition, undernourished children are at risk of long‐term developmental delays and have a 5‐to 20‐fold higher mortality rate than well‐nourished children (Mwene‐Batu et al. 2020). It can be the primary cause of a child's death or a contributing factor that markedly increases the mortality rate in children with common childhood illnesses, such as pneumonia, measles, or diarrhea. Globally, acute malnutrition affects 45 million children under the age of five. The vast majority of these cases, approximately 97% of cases of acute malnutrition, are found in Asia and Africa (United Nations Children's Fund (UNICEF), World Health Organization (WHO), & International Bank for Reconstruction and Development/The World Bank 2023).

Children with acute malnutrition were treated as inpatients for many years until the United Nations (UN) and World Health Organization approved the community‐based management of acute malnutrition program in 2007 (World Health Organization 2020). In Ethiopia, the Community‐based Management of Acute Malnutrition Program was launched in 2000. By 2008, it was incorporated into the Health Extension Program (Lemma et al. 2012). Less than 20% of children suffering from severe acute malnutrition (SAM) globally have access to the treatment they need (Rogers et al. 2015). Countries with a high burden of acute malnutrition are usually those where access to effective treatment is most limited and the availability of necessary resources is scarce (Hobbs et al. 2014). As a result, to improve the coverage of screening for wasting and ultimately identify more children with wasting for early referral, a Family Mid Upper Arm Circumference (MUAC) approach was developed, in which mothers, fathers, and other caregivers are trained to identify wasting in their children using MUAC tape (Buttarelli et al. 2020).

The ‘Family MUAC’ approach, also known as MUAC for mothers or Mother‐MUAC, equips mothers and caregivers to detect early signs of malnutrition in children using a simple MUAC tape. This method allows families to identify malnutrition as effectively as Community Health Workers (CHWs), leading to earlier detection and reduced hospitalizations. It improves children's health management and frees CHWs to focus on additional responsibilities. Family MUAC has the potential to increase early case detection and improve coverage for acute malnutrition (Majiwa et al. 2024). It was first piloted by the Alliance for International Medical Action in Niger in 2012 (Blackwell et al. 2015). The Family MUAC approach is still being piloted in most African countries, but there is still a need to integrate it into primary healthcare systems and standardize the indicators for monitoring and evaluation (Majiwa et al. 2024).

Therefore, this study aimed to evaluate the impact of Family MUAC on severity levels at admission and further quantify the proportions of moderate acute malnutrition (MAM) and SAM among children referred to health posts by mothers compared with those referred by health extension workers (HEWs).

## 2 Methods and Materials

### 2.1 Study Area/Setting

The study was conducted in Chiro and Gemechis District of West Hararghe zone. Many lowland regions of Oromia, such as West Hararghe, experience rainfall shortages. One of the locations with a high prevalence of food insecurity is Chiro District. In Chiro District, the main sources of income are the production of cereal crops along with cash crops, particularly khat.Chiro and Gemechis Districts are one of the Districts that are frequently affected by drought. There is high prevalence of under nutrition among children in West Hararghe zone, Cirro District, since the area is drought‐prone area. It is also an area where the family MUAC approach has been implemented for more than a year.

The 2007 national census reported a total population for this District of 294,295, of whom 150,917 were men and 143,378 women; whereas Gemechis district have 235,638 total populations of which 119,485 are males and 116,153 are females in 2019. In Gemechis District there are around 33,008 under 5 children whereas in Chiro District approximately there are 21,978 under five children.

### 2.2 Study Design

We conducted a retrospective longitudinal study to quantify the effect of employing the Family MUAC approach in identifying the severity of acute malnutrition at admission among children aged 6–59 months who were admitted to a health post. This study was conducted from February to May 2024.

### 2.3 Sample Size Determination and Participants Selection

To assess the effectiveness of family MUAC measurement in identifying the severity of acute malnutrition at admission, the sample size was calculated based on the mean difference formula. The sample size of 336 (168 study participants in each group) was estimated using the formula for mean difference with the following assumptions: expected difference in mean MUAC upon admission between the Mother‐Referral and HEWs‐Referral groups at 0.4 cm and standard deviation (SD) of 1.3 cm (12), 95% confidence level, and 5% margin of error. The mean MUAC values obtained from a study conducted in Mali served as essential parameters for calculating the sample size.

During the study period, all children aged 6–59 months and their caregivers visiting the health post were invited to participate. Those referred to the health post by their mothers were classified as the Mother (Caregivers)‐Referral Group, while children referred by HEWs were categorized as the HEW‐Referral Group. Hews‐Referral. Enrollment continued until the required sample size was achieved.

In the study districts, we first identified two health posts that received referrals from the mothers or caregivers. Approximately 8–10 months before the study, over 487 mothers in the selected kebeles (‘kebeles’ refers to the smallest administrative units in Ethiopia, similar to neighborhoods or communities. They serve as the local level of governance and are responsible for various administrative functions, including community health services) were given training on the Family MUAC approach. This training covered the practical aspects of MUAC measurement and its role in diagnosing and managing malnutrition, with an emphasis on early detection to reduce the mortality risk.

Mothers were provided with color‐banded MUAC tapes post‐training and given opportunities to practice measuring their children's MUAC and identifying signs of edema. The color‐banded design of the MUAC tape allows for easy interpretation of measurement results, enabling mothers to effectively assess the nutritional status of their children.

### 2.4 Variables

#### 2.4.1 Exposure Variable

##### 2.4.1.1 Source of Referral

The exposure variable in this study was the source through which the children were screened and referred to health posts. The source of referral could be either mothers (Mother‐Referral group) or health Extension Workers (HEWs‐Referral group). HEWs refer children identified as having MAM or SAM during their monthly community outreach activities to health posts for admission and treatment. The source of referral was used to classify the study participants into the Mother‐Referral and HEWs‐Referral groups. This classification was based on information obtained retrospectively through structured interviews with the child's mother or caregiver enrolled in the study. Participants were classified into ‘mother's referral’ and ‘HEWs referral’ groups based on retrospective information obtained from patient records and caregiver interviews. Specifically, we collected data on the sources of referral for each child, identifying whether they were referred by their mothers or HEWs. To ensure the accuracy and reliability of this categorization, we reviewed health post registers to verify the source of referral and conducted structured interviews with caregivers to identify the relevant information. Children referred to the health post based on mother/caregiver screening were categorized into the Mother‐Referral group. Children who were referred to a health post based on HEW screening were categorized into the HEWs Referral group.

#### 2.4.2 Outcome Variable(s)

Level of severity at admission: To determine the severity of malnutrition upon admission, trained HEWs assessed the MUAC of all children enrolled in the study at admission. The severity level at admission was classified into two categories: children with a MUAC measurement below 11.5 cm were categorized as having severe acute malnutrition, indicative of a critical nutritional status, while those with measurements between ≥ 11.5 ≤ 12.5 cm were classified as having moderate malnutrition, signaling a less severe case.

### 2.5 Covariates

#### 2.5.1 Demographic and Socioeconomic Characteristics

Sociodemographic and economic characteristics, such as age, sex, religion, level of education and wealth status, household food insecurity, and distance from the health center/health post, were assessed using interviewer‐administered questionnaires.

The age of caregivers was recorded in complete years, while the age of children was recorded in months.

**The education levels** of mothers, classified using scales ranging from no education to secondary education and above, were assessed.

**The wealth index** was calculated using data from household surveys, which involved selected variables related to asset ownership, housing characteristics, and access to basic services, and was transformed into numerical value. Principal component analysis (PCA) was used to generate wealth status. It includes several socioeconomic factors that are used to compute the wealth index. The results were divided into categories representing the poorest, poor, medium, rich, and richest households.

**Household food insecurity** was assessed using the Household Food Insecurity Access Scale (HFIAS), which contains nine occurrence and nine frequency questions assessing whether the condition occurred rarely (once or twice), sometimes (three to ten times), or often (more than ten times) in the past 4 weeks. Based on this score, participants were categorized as food secure, mildly food insecure, moderately food insecure, or severely food insecure (Coates et al. 2007).

**Vaccination status** was classified as fully, partially, or not vaccinated. Fully vaccinated children were those who had received all vaccines recommended for their age group (e.g., measles vaccine at 9 months and the second dose at 15 months, DPT vaccine at 6, 10, and 14 weeks, etc.) or a child who met the vaccination milestone based on the EPI schedule. Partially vaccinated children missed at least one vaccine appropriate for their age. And not vaccinated for children who had never been vaccinated **Vaccination status** was assessed based on the receipt of one dose of BCG vaccine, three doses of diphtheria‐tetanus‐pertussis‐hepatitis B‐Haemophilus influenza type b (DTP3‐HepB‐Hib) pentavalent vaccine, three doses of polio vaccine, and two doses of measles vaccine in children aged 12–23 months (Barrow et al. 2023).

To assess **infant and young child feeding (IYCF)** practices (Children 6–24 months), the study relied on several key indicators. These include early initiation of breastfeeding, which evaluates the proportion of infants breastfed within 1 h of birth. Exclusive breastfeeding for the first 2 days after birth was also assessed. Continued breastfeeding was measured to assess breastfeeding duration. Furthermore, the minimum dietary diversity was evaluated for the variety of foods consumed by infants and young children. The minimum meal frequency was used to assess the frequency of meals or feeds given to infants and young children within specific age ranges. Finally, the minimum acceptable diet (MAD), a composite indicator of dietary diversity and meal frequency, was assessed (World Health Organization & UNICEF 2021).

### 2.6 Data Quality Assurance

The data collectors received adequate training in the research objectives, methodology, and data collection approach. The data collection tools were translated into two local languages: Amharic and Afan‐Oromo. Survey questionnaires were designed to assess the socioeconomic status of the study participants, ensuring that the two groups were comparable. Additionally, the questionnaires included questions regarding the sources of referral and the results of anthropometric measurements.

We conducted a pretest and modified the tools based on the results of the pretesting. To ensure clarity, contextual relevance, and usability of the questionnaire, a pretest was conducted with 36 caregivers at a nearby non‐study health post. Based on their feedback, several revisions were made, including merging overlapping questions, clarifying terminology, simplifying response options, and adjusting question phrasing. A detailed summary of the pretest findings and corresponding modifications is provided in Supporting Information S1: Table 1.

A MUAC measuring tape for children with a threshold set at 11.5 cm was used to assess the children. HEWs received training on proper MUAC measurement techniques in accordance with the country's malnutrition management guidelines. The HEWs identified the midpoint of each child's upper arm, situated between the shoulder and elbow tips. They then wrapped the tape around the arm at this midpoint and recorded the MUAC measurements to the nearest 0.1 cm.

### 2.7 Data Management and Analysis

Data were collected using smartphones and the Kobo Toolbox (https://kc.kobotoolbox.org). The data were then exported to SPSS version 25 for cleaning and analysis. A descriptive analysis was conducted for socio‐demographic and economic factors, household food insecurity, child characteristics, health‐seeking behavior, and child feeding practice.

The HFIAS was used to measure the access component of household food insecurity. PCA was used to compute the wealth index of households' **dietary diversity for 6–23 months** was assessed based on the consumption of foods and beverages from at least five out of eight defined food groups during the previous day. **Meal frequency** was assessed based on the consumption of solid, semi‐solid, or soft foods (including milk feeds for non‐breastfed children) at least the minimum number of times during the previous day. **The MAD at 6–23 months** was assessed based on receiving at least the minimum dietary diversity and minimum meal frequency for their age during the previous day. In this study, we compared the MUAC measurements of children referred by mothers or caregivers with those taken by trained HEWs at the time of admission. Mothers or caregivers reported the color‐coded MUAC classification for their child, while HEWs confirmed the MUAC measurements using a standardized number‐banded MUAC tape. This admission measurement served as a point of comparison with the MUAC values of children referred by. Chi‐square test at 95% CI was used to assess whether there was a difference in wealth, HH food insecurity, child feeding practices, and health‐seeking behavior among the exposed and nonexposed groups. The distribution of the median MUAC at admission in both groups was visualized using a box plot.

Additionally, a multivariable logistic regression model was fitted to estimate the impact of the Family MUAC approach on the reduction in malnutrition severity at admission. The model also estimated the effect of Family MUAC on the proportion of children admitted with MAM and SAM. Both models were adjusted for potential confounding variables.

#### 2.7.1 Ethics Statement

Ethical clearance was obtained from Addis Ababa University, College of Health Science, School of Public Health, and a permission letter to carry out the study was obtained from Hararghe zone regional bureau. All study participants were given detailed information about the aims and methods of the study before the interview, and informed verbal consent to participate in the current study was obtained. The information obtained from the study was used only for the purpose of study, and the recorded data wasn't accessed by a third party except the principal investigator, and confidentiality was kept.

## 3 Results

### 3.1 Socio Demographic and Economic Characteristics

A total of 336 mothers/caregivers were enrolled in the study, with 118 participants each in the Mother‐Referral and HEWs‐Referral groups. Among them, 324 were biological mothers, and 12 were caregivers. The median age of the mothers and caregivers was 31 years (range, 18–48 years).

We found that the majority of households and children in the Mother‐Referral group had smaller household sizes (five or fewer members), higher wealth status, lived closer to health facilities, were fully vaccinated, received Vitamin A doses within the last 6 months, used water from protected sources, practiced good solid waste management, maintained good handwashing practices, and exhibited better healthcare‐seeking behavior for common childhood illnesses. In contrast, households in the HEWs‐Referral group were more food‐secure than those in the Mother‐Referral group. However, no significant differences were found between the Mother‐Referral and HEWs‐Referral groups in terms of breastfeeding (IYCF) indicators (Table 1).

**TABLE 1:** Socioeconomic characteristics, dietary diversity, hygiene, and environmental sanitation related characteristics in West Haraghe in accordance with their referral type.

Characteristics	Mother‐referral	(HEW's referral)	*p*‐value
Birth order			< 0.001
1st	50 (29.7%)	41 (24.4%)	
2nd –4th	114 (67.8%)	103 (61.3%)	
≥ 5th	4 (2.5%)	24 (14.3%)	
Vaccination status			0.016
Fully vaccinated	132 (78.6%)	111 (66.1%)	
Partially vaccinated	15 (8.9%)	16 (9.5%)	
Not vaccinated	21 (12.5%)	41 (24.4%)	
Had taken vitamin A dose in the last 6 months			0.018
Yes	148 (88.1%)	130 (77.4%)	
No	20 (11.9%)	38 (22.6%)	
Deworming			0.093
Yes	110 (65.7%)	95 (56.5%)	
No	58 (34.3%)	73 (43.5%)	
Had diarrhea at least once in the past 14 days			0.1
Yes	10 (6%)	8 (4.8%)	
No	158 (94%)	160 (95.2%)	
Had cough, fever, and shortness of breath in the past 14 days			0.15
Yes	7 (4.2%)	5 (3%)	
No	161 (95.8%)	163 (97%)	
Exclusively breastfed for the first two days after birth			0.46
Yes	37 (71.2%)	28 (63.6%)	
No	15 (28.8%)	16 (36.4%)	
Minimum meal frequency (%)			0.14
Adequate	19 (36.5%)	10 (22.7%)	
Inadequate	33 (63.5%)	34 (77.3%)	
Minimum acceptable diet			0.08
Adequate	11 (23.4%)	4 (9.5%)	
Inadequate	36 (76.6%)	38 (90.5%)	
Minimum dietary diversity			0.1
Adequate	15 (31.3%)	10 (20.8%)	
Inadequate	33 (68.7%)	38 (79.2%)	
Source of drinking water			0.011
Protected	156 (92.9%)	141 (83.9%)	
Unprotected	12 (7.1%)	27 (16.1%)	
Availability of latrine			0.06
Yes	128 (76.2%)	113 (67.3%)	
No	40 (23.8%)	55 (32.7%)	
Hand washing practice			0.02
Good	104 (61.9%)	83 (49.4%)	
Poor	64 (38.1%)	85 (50.6%)	
Solid waste management practice			0.01
Good	57 (33.9%)	40 (23.8%)	
Poor	123 (74.1%)	116 (69.2%)	
Seeking treatment for the sick child			< 0.001
Yes	119 (70.8%)	82 (48.8%)	
No	49 (29.2%)	86 (51.2%)	
Care sought for the child's illness			0.06
Public Sector (Government Hospital, Health Center, and Health Post)	115 (96.66%)	82 (100%)	
NGO (Health facility or Other NGO)	3 (2.5%)	0	
Shop or Drug Vendor	1 (0.84%)	0	
Time of health facility visited			0.0001
On the first day	89 (74.8%)	68 (82.9%)	
Within first week	27 (22.7%)	9 (11%)	
Within second week	3 (2.5%)	5 (6.1%)	
Reason for visiting health facility			0.0001
Child's condition worsened	62 (52.1%)	18 (22%)	
In‐order not the child's condition get worsened	57 (47.9%)	62 (75.6%)	
Other people advise	0	2 (2.4%)	
Reason for not visiting health facility			0.001
Distance from health facility	16 (32.6%)	36 (41.9%)	
Lack of money	29 (59.2%)	43 (50%)	
Illness was not serious	4 (8.2%)	5 (5.8%)	
Mothers being busy	0	2 (2.3%)	

### 3.2 Screening

**FIGURE 1:**
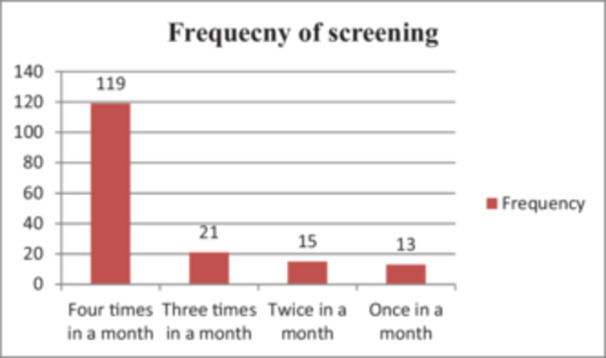
Frequency of screening mid‐upper arm circumference (MUAC) by mothers/caregivers who were trained on MUAC tape in Chiro and Gemechis Districts.

A total of 487 mothers and caretakers were trained to screen children by MUAC color‐coded class and check for edema during the initial training sessions in the two districts. Of the 336 study participants, 168 (50%) had received training on how to use MUAC tape, and the majority of them, 149 (88.7%), had received at least one refreshment training. Of these 168 participants, 86(51.2%) mentioned that they had received the training 1 year ago, while the remaining 48.8% had attended the training 8–10 months prior. 81(48.2%) participants reported that their family members had also attended training on the utilization of the MUAC tape, and 91.7% of them had visited the health post based on the MUAC screening result. Regarding the frequency of screening using the MUAC tape, the majority 119(70.8%) reported that they screened their child four times a month (Figure 1).

### 3.3 Agreement and Accuracy in MUAC Measurements by Caregivers and Health Workers

We observed that 93.2% of MUAC measurements and self‐referrals by mothers/caregivers based on MUAC color were accurately confirmed by HEWs assessing the same children at admission. Specifically, measurements by caregivers/mothers showed a sensitivity and specificity of > 80% and > 93.9%, respectively, in classifying SAM status in their children. Correlation analysis between MUAC measurements by mothers/caregivers and HEWs at admission revealed a significant correlation (*p* < 0.0001) and a perfect positive linear relationship (Pearson's correlation coefficient *r* = 1), indicating a strong agreement and consistency between the measurements.

### 3.4 Source of Referral and Level of Severity of Acute Malnutrition at Admission

The median MUAC at admission was 119 mm (IQR116–120) in the Mother‐Referral group (referral by mothers/caregivers using Family MUAC) and 116 mm (IQR, 115–119) in the HEWs‐Referral group (screened and referred by HEW's).

**FIGURE 2:**
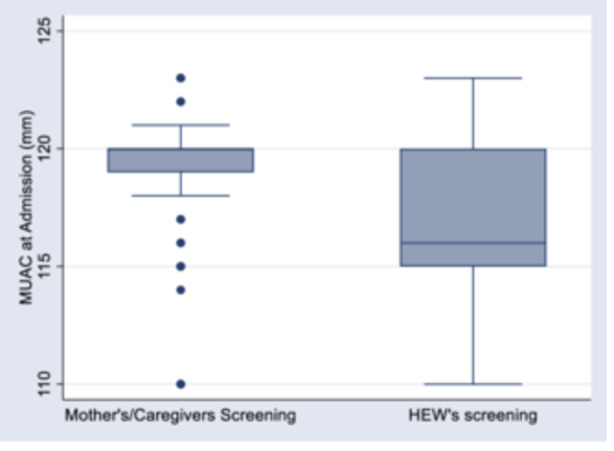
Distribution of MUAC at admission among children aged 6–59 months in mothers screening and HEWs screening. HEWs, Health Extension Workers; MUAC, mid‐upper arm circumference.

**FIGURE 3:**
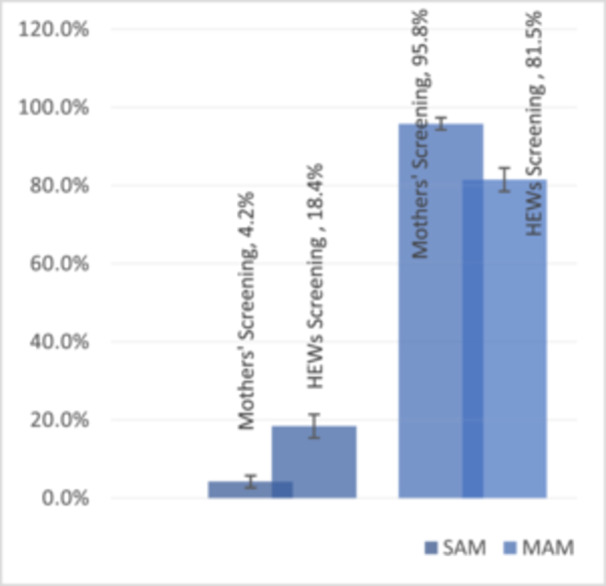
Proportion of MAM and SAM among children at admission referral type. MAM, moderate acute malnutrition; SAM, severe acute malnutrition.

Further analyses were conducted to compare the proportion of children admitted with MAM and SAM between those referred by mothers/caregivers and HEWs. Our findings revealed that 4.2% of the children referred by mothers/caregivers were diagnosed with SAM, compared to 18.4% of those referred by HEWs. Conversely, 95.8% of children referred by mothers/caregivers were identified as having MAM, whereas this proportion was 81.5% among those referred by HEWs (Figure 2) and (Figure 3).

### 3.5 Effect of Source of Referral on the Level of Severity of Acute Malnutrition at Admission

Table 2 shows the multivariable regression analysis model for the effect of Family MUAC measurements in identifying the severity of acute malnutrition at admission among children aged 6–59 months. Multivariable logistic regression revealed a significant effect of screening by mothers/caregivers on the severity level at admission. After accounting for confounding variables, children referred through Mother/Caregivers Screening were significantly less likely to have severe MUAC at admission than those referred through HEW screening. The risk among the exposed group (referred to as mothers/caregivers) was reduced by approximately 81% compared to those referred by HEWs. Specifically, children referred through mothers/caregivers' screening are 0.19 times as likely to have severe MUAC at admission compared to those referred by HEWs, indicating that the risk of severe MUAC at admission is approximately 81% lower for children referred through mothers/caregivers' screening (ARR = 0.19; risk reduction = 1–0.19 = 0.81 or 81%).]

**TABLE 2:** Multivariable regression analysis model for the effect of Family MUAC approach on the level of severity at admission among children aged 6–59 months.

	Level of severity at admission		Bivariate CRR (95% CI)	*p*‐value	ARR (95% CI)	*p*‐value
Characteristics	Severe	Not severe				
Referral type						
Mothers	20 (25%)	148 (57.8%)	0.24 (0.13,0.42)	< 0.001	0.19 (0.06, 0.59)	
HEWs	60 (75%)	108 (42.2%)	1.0		1.0	< 0.0001
Sex of caregiver						
Male	2(2.5%)	6 (2.3%)	1	0.936		
Female	78 (97.5%)	250 (97.7%)	1.61 (0.29–8.89)			
Age of caregiver (years)				0.055		0.06
18–29	17 (21.3%)	93 (36.3%)	1			
30–35	44 (55%)	130 (50.8%)	3.15 (1.465–6.77)		2.8 (1.2–6.5)	
> 35	19 (23.7%)	33(12.9%)	1.70 (0.879–3.29)		1.5 (0.7–2.8)	
Wealth index				0.07		< 0.0001
Poorest	70 (87.5%)	47 (18.4%)	1			
Poor	8 (10%)	96 (37.5%)	1.031 (0.15–12.48)			
Medium	2 (0.25%)	86 (33.6%)	1.38 (0.116–16.58)			
Rich	0	19 (7.4%)	0.60 (0.49–0.64)		0.80 (0.41–0.92)	
Richest	0	8 (3.1%)	0.30 (0.23–0.52)		0.42 (0.34–0.72)	
Family Size				0.001		< 0.0001
> 5	46 (54.8%)	53 (21%)	1		1	
≤ 5	38 (45.2%)	199 (79%)	0.25 (0.13–0.47)		0.29 (0.15–0.55)	
Distance from health facility						< 0.0001
> 30 min	31 (38.7%)	42 (16.4%)	1	0.001		
< 30 min	49 (61.3%)	214 (83.6%)	0.086 (0.043–0.172)		0.088 (0.043–0.180)	
Health‐Seeking Behavior				0.011		0.010
Not seeking treatment for sick child	42 (52.5%)	93 (36.3%)	1		1	
Seeking treatment for sick child	38 (47.5%)	163 (63.7%)	0.51 (0.311–0.85)		0.59 (0.33–1.07)	
Recent medical illness				0.02		< 0.0001
Yes	18 (22.5%)	11 (4.3%)	1		1	
No	62 (77.5%)	245 (95.7)	0.43 (0.16–0.87)		0.54 (0.22–0.86)	
Dietary Diversity						
Dietary diversity score > 5	48 (60%)		1	0.9		
Dietary diversity score < 5	32 (40%)		0.98 (0.53–1.8)			

*Adjusted for potential confounders such as demographic factors (age and sex), socioeconomic indicators (household food insecurity, distance from health facility, family size, and wealth index), and child‐related factors (vaccination status, birth order, dietary diversity, and recent medical illness).

Children from wealthier households had a significantly lower risk of severe MUAC at admission than those from the poorest households. Specifically, children from wealthy households had a 20% lower risk of severe MUAC (ARR = 0.80, 95% CI: 0.41–0.92, *p* < 0.0001). The risk was even lower for children from the richest households, with a 58% lower risk of severe MUAC at admission (ARR 0.42, 95% CI 0.34–0.72, *p* < 0.0001).

Similarly, children from households with fewer than five members had a significantly lower risk of severe MUAC at admission than those from larger families. Specifically, children from smaller households had a 71% lower risk of severe MUAC (ARR = 0.29, 95% CI: 0.15–0.55, *p* < 0.0001).

Children living less than 30 min from a health facility also had a significantly lower risk of severe MUAC at admission than those living farther away. Specifically, children in closer proximity to a health facility had a 91.2% lower risk of severe MUAC (ARR = 0.088, 95% CI: 0.043–0.180, *p* < 0.0001).

Additionally, children whose caregivers sought treatment for sick children had a 41% lower risk of severe MUAC (ARR = 0.59, 95% CI: 0.33–1.07, *p* = 0.010). Finally, children without recent medical illnesses had a significantly lower risk of severe MUAC at admission than those with recent illnesses. Specifically, children without recent illnesses had a 46% lower risk of severe MUAC (ARR = 0.54, 95% CI: 0.22–0.86, *p* < 0.0001). However, minimum dietary intake, sex, and age of the caregiver were not significantly associated with the risk of severe MUAC at admission.

## 4 Discussion

This study used a longitudinal design to assess the impact of a family‐based MUAC approach on the severity of acute malnutrition at admission. Children were categorized based on whether they were referred for screening by their mothers/caregivers or HEWs. Trained HEWs measured MUAC upon admission to health posts. Our results indicate that children referred by mothers/caregivers had a lower risk of SAM upon admission than those referred by HEWs. Furthermore, the median MUAC at admission was higher in children referred by mothers/caregivers, suggesting earlier malnutrition detection.

This finding aligns with that of a study conducted in the Mirrah district of Niger, which reported a similar trend. In that study, the median MUAC at admission among mothers was 1.6 mm higher than that of CHWs. Additionally, children admitted upon referral by their mothers showed a reduced need for inpatient care. This consistency in findings further supports the notion that maternal screening and referral may lead to earlier detection of malnutrition than other screening methods (Alé et al. 2016). In this study, children in the HEWsgroup were more likely to have severe MUAC at admission, indicating that the risk of severe MUAC at admission was lower in children referred through mother/caregiver screening. Despite the absence of directly related studies, other research and field reviews support the role of Family MUAC measurements in the early detection of acute malnutrition.

Findings from a study conducted in Kenya suggest that this simplified tape has the potential to facilitate the early identification of MAM, as indicated by an increase in the average MUAC upon admission among children referred for maternal screening (Grant et al. 2018). In another study conducted in Burkina Faso to assess the impact of the Family MUAC on recovery rates, it was shown that the Family MUAC leads to early detection (Daures et al. 2020). This can be attributed to the higher frequency of screening conducted by mothers (four times a month), which enabled earlier detection with higher MUAC levels upon admission.

This study also revealed a significant difference in the proportion of children admitted with MAM and SAM between those referred by mothers/caregivers and HEWs. Children referred by a mother or caregiver (the exposed group) showed a significantly lower likelihood of being admitted for SAM than the HEWs‐Referral group. This indicates that the risk of SAM admission was lower in the Mother‐Referral group than in the HEWs‐Referral group. A similar finding was reported in a study conducted in Niger, indicating a higher proportion of MAM among mothers [ARR (0.88, (95% CI = 0.80, 0.96)](9). This finding is also consistent with a study conducted in Mali and Chad, which showed a higher rate of MAM among self‐referrals through maternal screening (Gnamien et al. 2021). This can be attributed to the timely identification of cases among mothers facilitated by regular screening using the MUAC tape.

The observed association between referral sources and admission severity may be due to various underlying mechanisms. One such mechanism could be the frequency of screening, which might affect the level of severity at admission. In this study, the majority (70.8%) screened their children four times a month, which led to the prompt identification of acute malnutrition. Thus, factors such as mothers' education, awareness of the importance of malnutrition screening and MUAC measurements, health‐seeking behavior, and proximity and accessibility to healthcare facilities could influence severity levels upon admission. Additionally, family support and perceived barriers, such as time constraints and competing priorities, could hinder maternal screening, thereby influencing the severity levels observed at admission (Lope et al. 2022). Further exploration of these mechanisms is necessary to guide targeted interventions aimed at enhancing referral practices and optimizing child health outcomes in similar contexts.

The use of multivariate analyses to account for potential confounding variables is one of the strengths of this study. Using this method, the study is better able to determine and assess the independent effects of the major independent variables on the desired outcomes. Multivariable logistic regression analysis was used to assess the impact of Family MUAC measurement on the severity of acute malnutrition among children aged 6–59 months. Notably, children referred through maternal or caregiver screening were significantly less likely to present with severe MUAC at admission than those referred by HEWs. Specifically, the risk of severe MUAC was reduced by approximately 80.5% for children referred by mothers or caregivers, indicating the critical role of family involvement in the early identification of malnutrition.

Several demographic and socioeconomic factors significantly influenced the severity of malnutrition at admission, regardless of the screening method. Children from wealthier households had a substantially lower risk of severe MUAC at admission than those from the poorest households. This finding aligns with previous studies that demonstrated that higher‐income families exhibited more proactive health‐seeking behaviors. As some healthcare expenses are covered out‐of‐pocket, a family's socioeconomic status plays a crucial role in determining their ability and willingness to seek timely medical care for their children (Mandlik et al. 2017).

Children from households with fewer than five members had a significantly lower risk of severe MUAC at admission than those from larger families. This may be attributed to the ability of parents in smaller families to allocate more resources, such as time and money, to the care of their sick child (Astale and Chenault 2015). In contrast, households with a higher number of children may struggle to provide adequate care and attention to each child, potentially increasing the risk of severe malnutrition (Yisak et al. 2015).

Children living less than 30 min from a health facility had a significantly lower risk of severe MUAC at admission than those residing farther away. Care‐seeking decisions are influenced by various factors; however, the proximity of healthcare facilities appears to be the most critical determinant. Long distances pose a significant barrier to accessing healthcare services, a challenge further exacerbated by the high poverty levels among slum residents, which limit their ability to afford transportation costs. Supporting this, a previous study conducted in a rural community in Kenya revealed that nearly a third (29.2%) of respondents indicated that they would have sought professional medical services if a healthcare center had been closer (Mbagaya et al. 2005).

Additionally, children whose caregivers sought treatment for sick children had a lower risk of severe MUAC at the time of admission. This could be explained by the fact that caregivers who promptly took their children to health facilities were likely to have better knowledge and awareness of malnutrition, including its symptoms and signs (Lyimo et al. 2024). In contrast, decisions regarding early treatment for severe acute malnutrition or other illnesses are often influenced by low levels of awareness and limited understanding of acute malnutrition in children under five (de Lima et al. 2020). This finding aligns with previous studies that highlighted how a caregiver's understanding of the risks associated with a disease can significantly motivate or discourage them from seeking early treatment for the patient. Limited information on childhood illnesses, including acute malnutrition, and insufficient coverage of malnutrition services may further exacerbate delays in seeking care.

A limitation may be measurement error, which can severely compromise the precision of MUAC measurements and result in an inaccurate assessment of the nutritional status of children. Inaccuracies in measurement techniques, especially when performed by HEWs in health posts, are a source of measurement errors. Measurement errors can occur even with training and standard protocols because of variations in how the MUAC tape is positioned, tension is applied inconsistently, or measurement landmarks are misinterpreted.

Additionally, the generalizability of the study findings may be limited as the study focuses on the West Haraghe Zone of Oromia Region, Ethiopia. As the research was conducted in a specific geographical region, it raises questions about the extent to which the study participants represented a broader population. This uncertainty arises because the population in West Haraghe may differ from that in other regions, affecting the applicability of the findings to other populations or settings. For instance, since the region experiences drought and higher rates of food insecurity, these factors may affect the generalizability of the results of this study.

Sampling bias may have occurred as a result of the use of consecutive sampling methods. As consecutive sampling involves selecting participants who are readily available or easily accessible, typically based on their sequential arrival or availability during the study period, biases can be introduced that compromise the representativeness of the study sample and the generalizability of the findings. A significant limitation of this study was recall bias. It relies on participants' past events and experiences, which may lead to inaccuracies or inconsistencies in the data, compromising reliability and validity.

In conclusion, involving mothers and caregivers in screening and referral processes significantly impacts the severity level at admission, with early detection correlating with reduced hospitalization and healthcare costs, timely intervention, mitigated severity and complications, and improved child health outcomes. The Family MUAC approach emphasizes the crucial role of families in healthcare, promoting community engagement and decentralization of healthcare services. Expanding the Family MUAC approach in areas with a high malnutrition caseload can aid in early detection and reduce morbidity and mortality in young children, and integrating it into existing healthcare services will ensure better coverage. The FMOH should emphasize and incorporate Family MUAC into current healthcare systems, particularly for children under the age of five. Future research should investigate the long‐term impact and effectiveness of Family MUAC screening on child health outcomes, including its potential to lower malnutrition‐related morbidity and mortality rates in diverse settings.

## Author Contributions

Meron Tamirat and Seifu Hagos Gebreyesus conceived the study. Meron Tamirat analyzed the data and wrote the first draft with input from Seifu Hagos Gebreyesus, Alinoor Mohammed Farah, Gudina Egeta, and Samson Gebremedhin. All authors have read and approved the final manuscript.

## Conflicts of Interest

The authors declare no conflicts of interest.

## Supporting information

Supporting Material Summary of Pretesting and Tool Modification.

## Data Availability

The data that support the findings of this study are available on request from the corresponding author. The data are not publicly available due to privacy or ethical restrictions.
