# Supplementary material for: Effect of Family MUAC Utilization in Identifying Severity of Acute Malnutrition at Admission to Nutrition Programs Among Children Aged 6–59 Months Ethiopia
Source: Matern Child Nutr. 2025 May 29;21(4):e70054. doi: 10.1111/mcn.70054 (PMC12454198; doi:10.1111/mcn.70054)
Supplement: Supplementary file 1 — Supporting Material Summary of Pretesting and Tool Modification. [file MCN-21-e70054-s001.docx]

**Supplementary Material: Summary of Pretesting and Tool Modification**

To ensure the clarity, relevance, and contextual appropriateness of the data collection tool, a pretest was conducted on an earlier draft version of the questionnaire. Pilot testing of the questionnaire was performed on 10% of the sample, which was not included in the actual study, and the questionnaire was modified based on the findings of the pilot study. The pretest involved 36 caregivers in a nearby non-study health post with characteristics similar to those of the study area. The feedback obtained from the pretest was used to revise and finalize the questionnaire. The following is a summary of the changes made as a result of the pretest.

Supplementary Table 1 Changes made to the questionnaire.

| **Section** | **Item/Question (with Response Options)** | **Feedback from Pretest** | **Action Taken** |
| --- | --- | --- | --- |
| Socio-demographic | Q104–105: 104. Have you ever attended school? (1. Yes 2. No) 105. What is the highest level of school you attended? (1. No education 2. Primary 3. Secondary 4. More than secondary) | Respondents were confused by the separation of the two questions. | Merged into one question asking about highest education level, including 'No education' option. |
| Child Health Characteristics | Q204: Who is the source of referral to the health post? (1. Mother's/caregiver's screening 2.HEW’s screening) | The purpose of the question was unclear to some respondents who did not understand that it aimed to identify whether the child was referred by the caregiver or by a HEW. | Clarified during training that this question seeks to determine if referral was based on caregiver's home screening or HEW's community screening. |
| Health-Seeking Behavior | Q404: When did you seek health care after the onset of the illness? (1. On first day  2.Within 1st week  3. Within 2nd week  4. After 2nd week) | Participants had difficulty understanding the time categories. | Clarified with examples, e.g., 'first day = same day symptoms started'. |
| Child Feeding | Q509–511: Types of solid, semi-solid or soft foods and liquids consumed by the child yesterday. | Respondents found the detailed food and drink recall questions long and challenging. | Grouped foods visually and used simplified language during interviews to support memory recall. |
| Household Food Insecurity | Q602, Q604, etc.: Frequency of food insecurity-related events. (1. Rarely 2. Sometimes 3. Often) | Terms like 'rarely', 'sometimes', and 'often' were not easily understood. | Provided clarifying examples (e.g., 'rarely = 1–2 times in 4 weeks') for each term. |
| Family MUAC Enrollment | Q704–705: 704. How many refresher trainings have you attended? 705. Is there anyone else who attended the training on MUAC measurement from your family members? (1. Yes 2. No) | Respondents often did not distinguish between initial and refresher trainings. The concept of 'refresher training' was unfamiliar, and many did not recall exact training frequency. Similarly, some misunderstood Q705 as asking whether other caregivers were currently using the MUAC tape. | Clarified the definition of 'refresher training' during data collection. Modified instructions to ask whether other household members were ever trained in any session, not just current users. |
| Family MUAC Enrollment | Q706: How often did you screen (Name) using MUAC tape? (1. Once a month  2.Twice a month  3. Three times a month 4. Four times a month  5. Five times or more) | Respondents found multiple frequency options difficult to differentiate. | Simplified categories to: 'once', 'twice', or 'more than twice a month'.  Response categories were restructured and read aloud more slowly with examples. Interviewers were trained to probe with contextual references (e.g., market days, HEW visits) to help caregivers recall screening frequency. |
